# Soft tissue re-growth after different crown lengthening techniques among Indian patients

**DOI:** 10.6026/973206300171130

**Published:** 2021-12-31

**Authors:** Siddharth Narayan, Arvina Rajasekar

**Affiliations:** 1 Department of Periodontology, Saveetha Dental College, Saveetha Institute of Medical and Technical Sciences, Chennai-600077, Tamilnadu, India

**Keywords:** crown lengthening, electrocautery, flap surgery, gingivectomy, laser, soft tissue regrowth

## Abstract

Patients often report complaining of fractured or decayed teeth with severe morphological deformities. However, all these clinical scenarios require the same level of care and consideration to rehabilitate form, function and esthetics. Some cases have sufficient clinical crown height while others often require an interdisciplinary approach in the form of orthodontic/surgical extrusion or surgical periodontal options. A common factor delaying treatment is soft tissue regrowth after crown lengthening which delays the impression required for final prosthesis. Therefore, it is of interest to compare the prevalence of soft tissue regrowth a week after different crown lengthening techniques including laser gingivectomy, electrocautery gingivectomy, modified Widman flap and apically repositioned. The parameters assessed included 1-week postoperative soft tissue regrowth after crown lengthening, age of patients and gender. It was observed that laser and electrocautery-assisted gingivectomy had a higher rate of soft tissue regrowth as compared to surgical techniques. It was further noted that laser and electrocautery assisted gingivectomy had a higher frequency of soft tissue rebound growth compared to surgical crown lengthening using modified widman flap and apically repositioned flap, which was statistically insignificant. Patients within the age groups of 26-60 years were found to have a higher tendency of soft tissue regrowth, which was found to be clinically and statistically significant (p<0.05).

## Background:

Crown lengthening is a surgical procedure designed to increase the extent of supragingival tooth structure for restorative or esthetic purposes by apically positioning the gingival margin removing supporting bone or both [1]. This procedure is done to restore esthetics, function and provide adequate tooth height (clinical crown height) for retention of prosthetic crowns. Various clinical factors are considered beforethe selection of an ideal technique to achieve the same such as presence of pockets, biological width, crown root ratio, periodontal status of adjacent teeth, smile line and extent of restorations on tooth [2]. Periodontology as a science has advanced considerably in terms of regenerative potential using platelet rich plasma, plasma rich in growth factors and platelet rich fibrin along with the use of herbal products as an arsenal against periodontal tissue destruction [3-6]. There is also evidence of potential of stem cells in periodontal regeneration currently being tried and tested in clinical trials. Parma Benfenati studies the effect of restorative margins coinciding with bone crest level in dogs and reported approximately 5mm of bone loss after 3 months of intervention [2]. Such findings were seen most often in clinical scenarios where the restorative margin encroached on junctional epithelium and supracrestal connective tissue. [7, 9] Periodontal literature has suggested ways of avoiding such scenarios by apically repositioned the flap to intentionally repositioned periodontal attachment apparatus in an apical position [10-12]. However long-term evaluation of apically repositioned flap showed a slight reduction in keratinised gingiva where gingivectomy was performed, suggesting apically repositioned flap does not result in a permanent shift of mucogingival junction [13]. Coronal displacement of gingival margin 12 months after surgical crown was seen by pontoriero in 2001 with postsurgical soft tissue regrowth bucco-lingually of 2.9 +/- 0.6 mm and proximally 1.2 =/- 0.7 mm [14]. This study however did not take into consideration the different techniques of crown lengthening, one of the few studies which compared the effect of apically repositioned flap and gingivectomy on biological width showed re-established biological width within 3 months with more preference toward apically repositioned toward apically repositioned flap [15]. Therefore, it is of interest to report data on the soft tissue re-growth after different crown lengthening techniques among Indian patients, which was the rationale behind present study.

## Methodology:

The current study was performed as a single centered retrospective university design, using dental information archiving software for the comparison of the entire patient outflow of Saveetha Dental College and Hospital, Chennai from 1st June 2019 till 1st March 2020. Two blinded investigators screened data, where inter-examiner agreement of ninety percent was reached prior to inclusion of individuals into the study. The segregation of data was initiated after ethical approval from Saveetha university scientific review board where patients included were individuals who underwent crown lengthening using laser gingivectomy, electrocautery gingivectomy, modified widman flap and apically repositioned flap.

Age group of patients was subdivided for analysis and interpretation into group A (14-25 years), group B (26-40 years), groups C (41-60 years) and group D (above 60 years). Similarly the region involved in the crown lengthening was subdivided into group A Upper arch, group B Lower arch, group C sextant 5 and group D sextant 2.Individuals excluded from this study were (i) pregnant women or lactating mothers (ii) smokers (iii) patients with uncontrolled systemic disease specifically affecting collagen metabolism (iv) patients under medication (v) Incomplete data collection (radiographs and periodontal status). The parameters assessed in this study included 1-week postoperative soft tissue regrowth after crown lengthening.

## Statistical significance:

All values obtained from this study were analysed using SPSS software version 23 (Statistical Package for the Social Sciences developed at the University of Stanford by Norman H. Nie, C. Hadlai Hull and Dale H.) where p values < 0.05 were considered to be statistically significant.

## Results & discussion:

**Figure 1:**
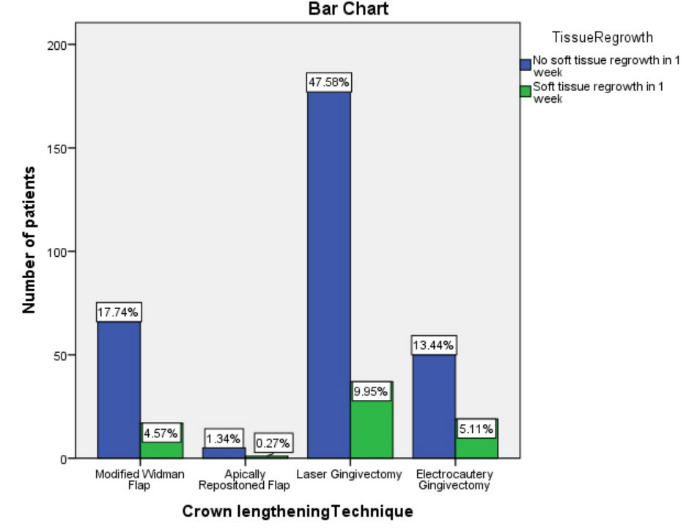
Illustration showing association between technique of crown lengthening and soft tissue regrowth; X-axis depicts treatment done; Y-axis depicts number of patients; Chi square test was done with a X2 value of 3.497, p value of 0.321, (p<0.05) considered statistically insignificant. There was an increased soft tissue regrowth after Laser gingivectomy, electrocautery gingivectomy and modified widman flap, which was clinically significant but statistically insignificant.

A total of 373 patients were included in this study 187 males and 186 females with different age groups 18-25 years (96), 26-40 years (167), 41-60 years (88) and above 60 years (22).(Table 1 - see PDF, graph 2) In this study group we observed that laser gingivectomy and electrocautery had a higher soft tissue regrowth after 1 week postoperative examination as compared to surgical techniques (Figure 1). It was observed that a higher tendency of soft tissue regrowth was seen among age groups of 26-40 years and 41 to 60 years (Table 1 - see PDF).

Among all findings obtained intragroup analysis was found to be statistically insignificant (p>0.05) using Chi square while parameters such as the relation between different age groups and soft tissue regrowth along with the crown lengthening technique employs and the presence of soft tissue regrowth 1 week postoperative were found to be significant with (p<0.05). We live in a day and age where aesthetic is considered as one of the key features that defines an individual's personality and bring out confidence, in terms of crown lengthening the use of procedures that leave scars such as conventional flap surgery are considered unaesthetic while laser assisted procedures, comprehensive procedures like lip repositioning and implant based full mouth rehabilitation in aggressive periodontitis cases are considered advantageous [16-18].

Soft tissue regrowth was seen with a higher tendency in laser gingivectomy and electrocautery gingivectomy groups.(Table 2 - see PDF) Both groups conserve bone leaving biological width as a sacrificeable tissue to obtain required clinical crown height. It is defined as dimension of space of root surface coronal to alveolar crest to which the junctional epithelium and connective tissue are attached (2.04 mm). Violation of biological width is associated with persistent gingival inflammation and tissue regrowth to correct this space occurring spontaneously within 3 months [15,19]. Similar studies by Pontoriero in 2001, demonstrated that over a 1 year follow up of healing following surgical crown lengthening, the marginal periodontium showed a tendency to grow more coronally from the level defined at surgery. This pattern of coronal displacement of the gingival margin was more commonly seen in patients with "thick" gingival tissue biotype [14].

**Figure 2:**
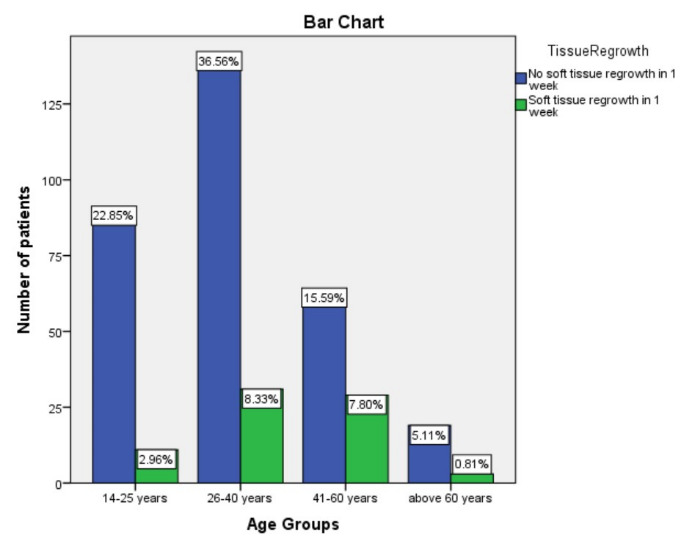
Bar graph shows association of age groups and soft tissue regrowth; X-axis depicts age groups in years; Y-axis depicts number of patients; Chi square test was done with a X2 value of 14.87, p value of 0.002, (p<0.05) considered statistically significant. There was an increase in soft tissue regrowth between age groups of 26 to 60 years, which was clinically and statistically significant.

The second finding of age groups 26-60 years having a higher frequency of soft tissue rebound regrowth could only be related to better wound healing after incomplete removal of connective tissue during the initial crown lengthening procedure.(Figure 2) All literature suggesting relation between age and periodontitis found increased age increased susceptibility to periodontitis with slower wound healing [20].

The limitations of this study include no exact measurement value of soft tissue regrowth to relate to demographic data or technique of crown lengthening. Another variable not included in the present study was the extent of crown lenghtening required for prosthetic rehabilitation of teeth, which changes depending on treatment planned and clinical visible crown height. The use of potential periodontal biomarkers of healing such as fibroblast growth factor 2 levels, cathepsin levels, endothelin 1 levels could have been correlated to obtained findings.[21-24]The present study population was not homogenous and different treatment techniques were applied to different number of patients with no correlation to oral hygiene status of the patients.

## Conclusion:

It was observed that laser and electrocautery assisted gingivectomy had a higher frequency of soft tissue rebound growth related to the age groups of 26-60 years as compared to surgical crown lengthening using modified widman flap and apically repositioned flap which was statistically significant (p<0.05).

## References

[1] https://members.perio.org/.

[2] http://www.quintpub.com/.

[3] Avinash K (2017). Int J Stem Cells..

[4] Panda S (2014). Contemp Clin Dent..

[5] Ramesh A (2016). J Intercult Ethnopharmacol..

[6] Ravi S (2017). Journal of Periodontology..

[7] Lanning SK (2003). J Periodontol..

[8] Kavarthapu A, Thamaraiselvan M (2018). Indian J Dent Res..

[9] Ramesh A (2016). Journal of Oral Biosciences..

[10] Nabers CL (1954). J Periodontol..

[11] Ramamurthy J, Mg V (2018). Asian J Pharm Clin Res..

[12] Priyanka S (2017). J Indian Soc Periodontol..

[13] Ainamo A (1992). J Clin Periodontol..

[14] Pontoriero R, Carnevale G (2001). J Periodontol..

[15] Ganji KK (2012). Int J Dent..

[16] Ramesh A (2019). J Indian Soc Periodontol..

[17] Ramesh A (2017). J Indian Soc Periodontol..

[18] Thamaraiselvan M (2015). J Indian Soc Periodontol..

[19] JG Maynard Jr, Wilson RD (1979). J Periodontol..

[20] Van der Velden U (1984). J Clin Periodontol..

[21] Khalid W (2017). Clin Diagn Res..

[22] Mootha A (2016). J. Int J Inflam..

[23] Khalid W (2016). Indian J Dent Res..

[24] Varghese SS (2015). Contemp Clin Dent..

